# AVD Annals of Vascular Diseases

**DOI:** 10.3400/avd.cv.21-01000

**Published:** 2021-12-25

## No. 1

### REVIEW ARTICLE

**1 The Primary Prevention of Venous Thromboembolism in Patients with COVID-19 in Japan: Current Status and Future Perspective**

Yugo Yamashita, MD, Norikazu Yamada, MD, and Makoto Mo, MD

### ORIGINAL ARTICLES

**5 Outcomes of Catheter-Directed Thrombolysis for Arteriovenous Fistula Thrombosis in Singapore: Is It Still Relevant Today?**

Clarice Biru Yeo, MBBS, Enming Yong, FRCSEd, MBA, Qiantai Hong, FRCSEd, Justin Kwan, FRCR, Lawrence Han Hwee Quek, FRCR, FRANZ, Uei Pua, FRCR, FSIR, Sundeep Punamiya, FAMS, Sadhana Chandrasekar, FRCS, Glenn Wei Leong Tan, FRCSEd, and Zhiwen Joseph Lo, FRCSEd, FACS

**11 Platelet Count Recovery after Endovascular Aneurysm Repair for Abdominal Aortic Aneurysm**

Kentaro Inoue, MD, PhD, Tadashi Furuyama, MD, PhD, Shun Kurose, MD, Shinichiro Yoshino, MD, Ken Nakayama, MD, Sho Yamashita, MD, Koichi Morisaki, MD, PhD, and Masaki Mori, MD, PhD

**19 Abdominal Aortic Neck Wrap for Refractory Type 1a Endoleak: A Case Series and a Novel Intraoperative Assessment Technique**

Ali Kordzadeh, MBBS, MSc, MD, FEBS, FEBVS, VA-BC, Tamer Sayed, MD, FEBVS, Manfred J. Ramirez, MSc, FRCS, Ioannis Prionidis, PhD, FRCS, Adam Howard, MBBS, BSc, MD, FRCS, and Tom Browne, FRCS

**23 A Pilot Study Investigating the Use of Regional Oxygen Saturation as a Predictor of Ischemic Wound Healing Outcome after Endovascular Treatment in Patients with Chronic Limb-Threatening Ischemia**

Takafumi Kayama, MD, Masaki Sano, MD, PhD, Kazunori Inuzuka, MD, PhD, Kazuto Katahashi, MD, PhD, Tatsuro Yata, MD, PhD, Yuta Yamanaka, MD, Ena Naruse, Naoto Yamamoto, MD, Hiroya Takeuchi, MD, PhD, and Naoki Unno, MD, PhD

**31 Lipoprotein Profiles before Heparin Administration in Patients with or without Coronary Thrombosis Following Atherosclerosis**

Tomomi Koizumi, MD, PhD, FACC, FESC, Hideaki Kaneda, MD, PhD, Nobuyuki Komiyama, MD, PhD, Ikuo Inoue, MD, PhD, Toshihiro Muramatsu, MD, PhD, and Katsuyuki Nakajima, PhD

**39 Carotid Stump Pressure and Contralateral Internal Carotid Stenosis Ratio During Carotid Endarterectomies: 1D-0D Hemodynamic Simulation of Cerebral Perfusion**

Sohei Matsuura, MD, Toshio Takayama, MD, PhD, Changyoung Yuhn, MS, Marie Oshima, PhD, Takuro Shirasu, MD, PhD, Takafumi Akai, MD, PhD, Toshihiko Isaji, MD, PhD, and Katsuyuki Hoshina, MD, PhD

**46 Use of Sponge-Foam Inserts in Compression Bandaging of Non-Healing Venous Leg Ulcers**

Rica Tanaka, MD, PhD, Hideaki Inoue, BS, Takeru Ishikawa, MS, Yuichi Ichikawa, MD, Rumiko Sato, MD, Azusa Shimizu, MD, PhD, and Hiroshi Mizuno, MD, PhD

### CASE REPORTS

**52 Successful Endovascular Repair of an Abdominal Aortic Aneurysm in a Patient with a Horseshoe Kidney and Accessory Renal Arteries Using an Aortic Cuff to Prevent Type II Endoleak**

Takuma Mikami, MD, Takeshi Kamada, MD, PhD, Hiroki Uchiyama, MD, Yosuke Kuroda, MD, PhD, Ryo Harada, MD, PhD, Syuichi Naraoka, MD, PhD, and Nobuyoshi Kawaharada, MD, PhD

**56 A Case of Human Immunodeficiency Virus-Positive Patient Diagnosed during the Treatment of Right Internal Iliac Pseudoaneurysm**

Yohei Iseki, MD, Masahiro Fujii, MD, PhD, Dai Nishina, MD, Shohei Mizushima, MD, Takahiko Mine, MD, PhD, Shin-ichiro Kumita, MD, PhD, Yosuke Ishii, MD, PhD, and Ryuzo Bessho, MD, PhD

**60 Hybrid Repair of Kommerell Diverticulum and Aberrant Subclavian Artery with Compressive Symptoms and a New Strategy: Case Report**

Junji Tsukagoshi, MD, Yutaka Iba, MD, PhD, Yoshihiko Kurimoto, MD, PhD, Ryushi Maruyama, MD, PhD, Yosuke Yanase, MD, PhD, Naritomo Nishioka, MD, PhD, Takahiko Masuda, MD, and Akira Yamada, MD, PhD

**64 Two Cases of Protruding Thrombus in the Ascending Aorta**

Noriyuki Abe, MD, Ken Yasumori, MD, Noriko Shimabukuro, MD, Takahiro Yamazato, MD, and Hiroshi Munakata, MD

**68 Aortic Dissection in Familial Patients with Autosomal Dominant Polycystic Kidney Disease**

Yu Inaba, MD, PhD, Motohiko Osako, MD, PhD, Michiko Aoki, NP, Mio Kasai, MD, and Kentaro Yamabe, MD

**71 Infected Aortic Aneurysm Caused by *Streptococcus zooepidemicus*: A Case Report and Literature Review**

Yuji Matsubayashi, MD, Noriyuki Takashima, MD, PhD, Yasuo Kondo, MD, Hodaka Wakisaka, MD, and Tomoaki Suzuki, MD, PhD

**75 A Successful Endovascular Technique for Complete False Lumen Thrombosis in Chronic Abdominal Aortic Dissection**

Hiromitsu Hiruma, MD, Yukihisa Ogawa, MD, PhD, Kiyoshi Chiba, MD, PhD, Takaaki Maruhashi, MD, Akiyuki Kotoku, MD, PhD, Hidefumi Mimura, MD, PhD, Takeshi Miyairi, MD, PhD, and Hiroshi Nishimaki, MD, PhD

**79 A Case of Isolated Infragenicular Arterial Lesions Successfully Treated with Open Endarterectomy**

Hiroko Okuda, MD, Shinsuke Kikuchi, MD, PhD, Atsuhiro Koya, MD, Hidehiro Takei, MD, PhD, and Nobuyoshi Azuma, MD, PhD

**83 Type B Acute Aortic Dissection as a Perioperative Complication after an Endovascular Abdominal Aortic Repair**

Kayoko Natsume, MD, PhD, Tsunehiro Shintani, MD, PhD, Masanori Hayahi, MD, Kazuhiro Ohkura, MD, PhD, Yuto Hasegawa, MD, and Takumi Ariya, MD

**88 Nutcracker Phenomenon with Menorrhagia in a Woman with Marfan Syndrome**

Kayo Sugiyama, MD, Toshiki Fujiyoshi, MD, Nobusato Koizumi, MD, PhD, and Hitoshi Ogino, MD, PhD

### ANNUAL REPORT

**92 The Outcomes of Endovascular Aneurysm Repair in Japan in 2017: A Report from the Japanese Committee for Stentgraft Management**

Katsuyuki Hoshina, PhD, Kimihiro Komori, PhD, Hiraku Kumamaru, PhD, and Hideyuki Shimizu, PhD

## No. 2

### ORIGINAL ARTICLES

Selection from the Japanese Journal of Phlebology 2019

**99 Deep Vein Thrombosis (DVT) Prophylactic Team Activity to Support DVT Prevention Protocol for the Purpose of the Prophylaxis of Pulmonary Thromboembolism (PTE) and Operation**

Shozo Tamura, BE, Mai Yamamoto, BN, Atsushi Kitagawa, MD, and Toshihiko Nagao, MD, PhD

**108 The Effectiveness of Endovenous Thermal Ablation for the Knee Symptoms of the Osteoarthritis with Varicose Veins**

Yuki Oga, MD, Satoru Sugiyama, MD, PhD, Susumu Matsubara, Yasuhiko Inaki, MD, Masashi Matsunaga, MD, and Akira Shindo, MD

**112 Clinical Results 5 Years after Great Saphenous Vein Stripping**

Hitoshi Kusagawa, MD, PhD, Yasuhisa Ozu, MD, Kentaro Inoue, MD, PhD, Takuya Komada, MD, PhD, and Yoshihiko Katayama, MD, PhD

### ORIGINAL ARTICLES

**118 Pattern of Chronic Venous Insufficiency among Patients Presenting to a Vascular Surgery Clinic in Low- to Middle-Income Countries (LMIC): A Cross-Sectional Study**

Zia Ur Rehman, MBBS, FCPS, ChM Vascular and Endovascular Surgery

**122 Optimal Duration of Compression Stocking Therapy after Endovenous Laser Ablation Using a 1470-nm Diode Dual-Ring Radial Laser Fiber for Great Saphenous Vein Insufficiency**

Shinsuke Mii, MD, PhD, Atsushi Guntani, MD, PhD, Ryosuke Yoshiga, MD, Takuya Matsumoto, MD, PhD, Eisuke Kawakubo, MD, PhD, and Jun Okadome, MD, PhD

**132 Ulnar-Basilic Arteriovenous Fistula for Hemodialysis Access: Utility as the “Second Procedure” after Radio Cephalic Fistula**

Shobhit Sharma, MS, MCh, Sudipta Bera, DNB, MCh, Vikas Deep Goyal, MS, MCh, Vivek Gupta, MS, MCh, and Navneeta Bisht, DNB

**139 Clinical Utility of the Candy-Plug Technique Using an Excluder Aortic Extender**

Yukihisa Ogawa, MD, PhD, Hiroshi Nishimaki, MD, PhD, Kiyoshi Chiba, MD, PhD, Tomotaka Iraha, MD, Takaaki Maruhashi, MD, Yuka Sakurai, MD, Takeshi Miyairi, MD, PhD, and Yasuo Nakajima, MD, PhD

**146 Anticoagulant Therapy for Cancer-Associated Venous Thromboembolism after Cancer Remission**

Nobuhiro Hara, MD, Tetsumin Lee, MD, PhD, Kentaro Mitsui, MD, Masashi Nagase, MD, Shinichiro Okata, MD, PhD, Giich Nitta, MD, Masakazu Kaneko, MD, Yasutoshi Nagata, MD, Toshihiro Nozato, MD, PhD, and Takashi Ashikaga, MD, PhD

**153 Novel Brain Protection Method for Zone 0 Endovascular Aortic Repair with Selective Cerebral Perfusion**

Ryuta Seguchi, MD, PhD, Ryuta Kiuchi, MD, PhD, Takafumi Horikawa, MD, Tatsuya Tarui, MD, PhD, Junichiro Sanada, MD, PhD, Hiroshi Ohtake, MD, PhD, and Go Watanabe, MD, PhD

### CASE REPORTS

**159 Rupture of Abdominal Aortic Aneurysm Caused by Combined Type IIIb and Type Ia Endoleak with the Endurant II Endograft: A Case Report**

Takanobu Okazaki, MD, Masaki Hamamoto, MD, PhD, Taiichi Takasaki, MD, PhD, Keijiro Katayama, MD, PhD, Taira Kobayashi, MD, and Shinya Takahashi, MD, PhD

**163 Successful Prophylactic Endovascular Therapy for a Rapidly Expanding Hepatic Arterial Aneurysm in a Patient with Vascular Ehlers–Danlos Syndrome**

Yukihiro Watanabe, MD, Koichi Akutsu, MD, PhD, Daisuke Yasui, MD, PhD, Fumie Sugihara, MD, PhD, Hideki Miyachi, MD, PhD, Hiroshi Hayashi, MD, PhD, Eiichiro Oka, MD, PhD, Hidenori Komiyama, MD, PhD, Shin-ichiro Kumita, MD, PhD, and Wataru Shimizu, MD, PhD

**168 Three Arterial Ruptures in a Patient with Neurofibromatosis Type 1**

Shotaro Higa, MD, Takaaki Nagano, MD, Junji Ito, MD, Akino Uejo, MD, Moriyasu Nakaema, MD, Yuya Kise, MD, PhD, and Satoshi Yamashiro, MD, PhD

**173 Late Vascular Injury at Both Edges of the VIABAHN Stent Graft after Endovascular Repair for Idiopathic Superficial Femoral Artery Rupture**

Yuta Tajima, MD, PhD and Kyosuke Kokaguchi, MD, PhD

**177 A Case of Thrombosed Inferior Mesenteric Artery Aneurysm Concomitant with Abdominal Aortic Aneurysm Treated by Endovascular Aneurysm Repair**

Yoichi Yamashita, MD, PhD, Sayako Nakagawa, MD, Kosuke Sakamoto, MD, Shohei Kitamoto, MD, and Taiko Horii, MD, PhD

**181 Total Debranching Plus Antegrade Thoracic Endovascular Aortic Repair without Side Clamping in a Patient with Arch Aneurysm and Ascending Aorta Calcification**

Ryutaro Isoda, MD, Yuji Kanaoka, MD, PhD, Tatsuya Watanabe, MD, PhD, Atsuhisa Ishida, MD, PhD, Masahiko Kuinose, MD, PhD, and Ichiro Morita, MD, PhD

**185 Successful Combined Treatment of Giant Carotid Body Tumor with Embolization Applied before Surgery**

Hakan Usta, MD, Izatullah Jalalzai, MD, Ferhat Borulu, MD, Eyupserhat Calik, MD, and Bilgehan Erkut, MD

**188 Various Neurological Symptoms Associated with Infected Internal Iliac Artery Aneurysm: A Case Report**

Kaoru Uchida, MD, Takashi Shuto, MD, PhD, Tomoyuki Wada, MD, PhD, Hideyuki Tanaka, MD, PhD, and Shinji Miyamoto, MD, PhD

**192 Shrinkage of a Giant Type-B Dissecting Aneurysm Treated by Complete False Lumen Occlusion 20 Years after Presentation: A Case Report**

Akimasa Morisaki, MD, PhD, Etsuji Sohgawa, MD, Yosuke Takahashi, MD, PhD, Hiromichi Fujii, MD, PhD, Yoshito Sakon, MD, PhD, Noriaki Kishimoto, MD, Kokoro Yamane, MD, and Toshihiko Shibata, MD, PhD

**198 A Case of Effective Balloon Fenestration for Localized Aortic Dissection Complicated with Acute Renal and Bilateral Limb Ischemia in a Thoracoabdominal Aortic Aneurysm**

Yusuke Imaeda, MD, Hiroyuki Ishibashi, MD, Yuki Orimoto, MD, Yuki Maruyama, MD, Hiroki Mitsuoka, MD, Takahiro Arima, MD, Ako Isogai, MD, Katsuhiko Matsuyama, MD, Kayo Sugiyama, MD, and Kojiro Suzuki, MD

### ANNUAL REPORT

**202 2018 JAPAN Critical Limb Ischemia Database (JCLIMB) Annual Report**

The Japanese Society for Vascular Surgery JCLIMB Committee , NCD JCLIMB Analytical Team

## No. 3

### ORIGINAL ARTICLES

**231 Digital Subtraction Angiography-Guided Foam Sclerotherapy with Polidocanol for Treating Superficial Venous Malformation**

Tuan Tran Anh, MD, PhD, Quyen Le Nguyen, MD, PhD, Quynh Mai Thi, MD, and Thong Pham Minh, MD, PhD

**236 Influence of Microalbuminuria on Long-Term Survival and Cardiovascular or Limb Events in Peripheral Arterial Disease**

Kuniki Nakashima, MD, PhD, Hisao Kumakura, MD, PhD, Ryuichi Funada, MD, PhD, Yae Matsuo, MD, PhD, Kimimasa Sakata, MD, PhD, Akiko Ichikawa, MD, PhD, Toshiya Iwasaki, MD, and Shuichi Ichikawa, MD, PhD

### CASE REPORTS

**244 Treatment of Coral Reef Aorta by Endovascular VIABAHN VBX Balloon-Expandable Stent-Graft Placement**

Kaoru Myouchin, MD, Katsutoshi Takayama, MD, Takeshi Wada, MD, Hidehiko Taguchi, MD, Toshihiro Tanaka, MD, and Kimihiko Kichikawa, MD

**249 Pleural Approach to Aberrant Right Subclavian Artery in Aortic Surgery**

Kazuhiro Kurisu, MD, Ken-ichi Imasaka, MD, PhD, Akira Hashino, MD, Yasutaka Ueno, MD, and Akira Shiose, MD, PhD

**252 Blue Rubber Bleb Nevus Syndrome Complicated by Enhanced-Fibrinolytic-Type DIC: A Case Report**

Shinya Yamada, MD, Masahisa Arahata, MD, Eriko Morishita, MD, PhD, and Hidesaku Asakura, MD, PhD

**256 Rapid Formation and Rupture of Multiple Abdominal Pseudoaneurysms: A Life Threatening Case of Segmental Arterial Mediolysis**

Ernest M Cheng, MD, Kerry L Chen, BMed, MD, Varsha Sharma, MBBS, FRACS, Juliana Yee, MBBS, Mark Power, BAppSc, MBBS, FRANZCR, Lubomyr D Lemech, MBBS (Hons), and Francis Chu, MBBS, FRACS

**260 In Situ Revascularization with a Rifampicin-Soaked Prosthesis to Treat Bare Iliac Artery Stent Infection: A Case Report**

Munetaka Hashimoto, MD, PhD, Yoshihisa Tamate, MD, PhD, Hiroko Sato, MD, PhD, Akihiko Murakami, MD, Shunsuke Shibuya, MD, PhD, and Naoki Yanagawa, MD, PhD

**264 A Case of Multilevel Aortic Disease Treated Using Cardiatis Multilayer Flow Modulator**

Mafalda Massara, MD, PhD, Antonino Alberti, MD, Andrea Cutrupi, MD, Vittorio Alberti, MD, Gaetana Franco, MD, and Pietro Volpe, MD

**267 Combination of Lymph Node Embolization and Musculocutaneous Flap Operation for Managing Groin Lymphorrhea**

Pham-Thi Viet Dung, MD, Nguyen Ngoc Cuong, MD, Thai Duy Quang, MD, Pham Hong Canh, MD, Le Tuan Linh, MD, and Nguyen Minh Duc, MD

**270 Ilio-Hepatic Artery Bypass for Hypoplasia of the Celiac Axis and Its Branches with an Inferior Pancreaticoduodenal Artery Aneurysm**

Masaru Nemoto, MD, PhD, Tatsuki Watanabe, MD, Yu Tadokoro, MD, Yutaka Takayama, MD, PhD, and Junji Yamamoto, MD, PhD

**273 Hyperbaric Oxygen Therapy Is an Effective Adjunctive Therapy to Manage Treatment-Resistant Venous Leg Ulcers**

Kotaro Suehiro, MD, Motoki Fujita, MD, Noriyasu Morikage, MD, Takasuke Harada, MD, Makoto Samura, MD, Ryo Suzuki, MD, Hiroshi Kurazumi, MD, Ryosuke Tsuruta, MD, and Kimikazu Hamano, MD

### HOW TO DO IT

**277 Bidirectional Sling Technique with Biopsy Forceps for Inferior Vena Cava Filter Retrieval**

Hitoshi Anzai, MD, PhD, Satoru Takaesu, MD, Tomoyuki Yaguchi, MD, Takayuki Shimizu, MD, Tatsunori Noto, MD, Yoshinori Nagashima, MD, and Naohiko Nemoto, MD

### ANNUAL REPORTS

**281 The Outcomes of Thoracic Endovascular Aortic Repair in Japan in 2017: A Report from the Japanese Committee for Stentgraft Management**

Katsuyuki Hoshina, PhD, Kimihiro Komori, PhD, Hiraku Kumamaru, PhD, and Hideyuki Shimizu, PhD

**289 Vascular Surgery in Japan: 2015 Annual Report by the Japanese Society for Vascular Surgery**

The Japanese Society for Vascular Surgery Database Management Committee Member, and NCD Vascular Surgery Data Analysis Team

**309 Erratum**

**312 Erratum**

## No. 4

### REVIEW ARTICLES

**315 Review on Disasters and Lower Limb Venous Disease**

Sergio Gianesini, MD, PhD, Erica Menegatti, PhD, Oscar Bottini, MD, and Yung-Wei Chi, MD

**323 Supplement of Clinical Practice Guidelines for Endovenous Thermal Ablation for Varicose Veins: Overuse for the Inappropriate Indication**

Makoto Mo, MD, PhD, Masayuki Hirokawa, MD, PhD, Hirono Satokawa, MD, PhD, Takumi Yasugi, MD, PhD, Takashi Yamaki, MD, PhD, Takaaki Ito, MD, Shiro Onozawa, MD, PhD, Takashi Kobata, MD, Nozomu Shirasugi, MD, PhD, Shintaro Shokoku, MD, PhD, Norihide Sugano, MD, PhD, Satoru Sugiyama, MD, PhD, Katsuyuki Hoshina, MD, PhD on behalf of Guideline Committee, Japanese Society of Phlebology, Tomohiro Ogawa, MD, PhD on behalf of Japanese Commitee of Endovenous Treatment for Varicose Veins

### ORIGINAL ARTICLES

Selection from Japanese Journal of Vascular Surgery 2020

**328 Evaluation of Perfusion Index as a Screening Tool for Developing Critical Limb Ischemia**

Nobuko Yamamoto, MD, Hideki Sakashita, MD, PhD, Noriyuki Miyama, MD, PhD, Kanako Takai, MD, and Hiroyoshi Komai, MD, PhD

Selection from the Journal of Japanese College of Angiology 2020

**334 Relationship between the Controlling Nutritional Status Score and Infrainguinal Bypass Surgery Outcomes in Patients with Chronic Limb-threatening Ischemia**

Satoshi Yamamoto, MD, PhD, Juno Deguchi, MD, PhD, Takuya Hashimoto, MD, PhD, Masamitsu Suhara, MD, PhD, and Osamu Sato, MD, PhD

### ORIGINAL ARTICLES

**341 Radiofrequency Ablation and Concomitant Sclerotherapy for the Treatment of Varicose Veins (VV): Perspectives from a Developing Country**

Muhammad Yousuf Memon, MBBS, DMRD, EDiR, FRCR (2a), VIR, Ilyas Sadiq, FRCS, FRCS (Intercollegiate Speciality Board), CCST, EITS, Safdar Ali Malik, MBBS, DMRD, Muhammad Bin Zulifqar, MBBS, FCPS, Muhammad Saad Malik, MBBS, and Muhammad Hammad Malik, MBBS

**348 Doppler is a Safe Criterion for Ensuring the Implementation of Eversion Carotid Endarterectomy**

Panagitsa D. Christoforou, MD, MSc, Chris N. Bakoyiannis, MD, PhD, Marianna Konidari, MD, Sotirios Georgopoulos, MD, PhD, and Thomas Kotsis, MD, PhD

**355 Cranial Tributary Ablation of the Saphenofemoral Junction during Laser Crossectomy of the Great Saphenous Vein**

Tsuyoshi Shimizu, MD, PhD, Yoshio Kasuga, MD, PhD, and Takeshi Shimizu, MD, PhD

**362 Distal Stent Graft-Induced New Entry after Total Arch Replacement with Frozen Elephant Trunk for Aortic Dissection**

Yoshikatsu Nomura, MD, PhD, Shuto Tonoki, MD, Motoharu Kawashima, MD, Jun Fujisue, MD, Gaku Uchino, MD, Shunsuke Miyahara, MD, PhD, Hiroshi Tanaka, MD, PhD, Tasuku Honda, MD, PhD, Nobuhiko Mukohara, MD, PhD, and Hirohisa Murakami, MD, PhD

### CASE REPORTS

**368 Iatrogenic Common Femoral Artery Occlusion Caused by a Suture-Mediated Closure System: A Case Report**

Nobuo Kondo, MD, PhD, Kensuke Oue, MD, Kohei Miyashita, MD, Satofumi Tanaka, MD, and Yoichiro Miyake, MD, PhD

**372 Aortic Root Replacement via Lower Hemisternotomy After an Esophageal Operation**

Kazuhiko Uwabe, MD, PhD and Noriyasu Masuda, MD, PhD

**376 Calciphylaxis After Aortic Valve Replacement in a Patient with End-Stage Renal Disease**

Kazunobu Hirooka, MD, PhD, Keisuke Anju, MD, Yoshihiro Moriyama, MD, Yuichi Araki, MD, Ekapot Bhunchet, MD, Ryoji Kinoshita, MD, Yohei Yamamoto, MD, Hidetoshi Uchiyama, MD, Masahiro Oonuki, MD, PhD, and Hiroyuki Tanaka, MD, PhD

**380 IgG4-Related Periaortitis Initially Suspected of Being an Aortic Intramural Hematoma in the Ascending Aorta**

Kazuki Takahashi, MD, Shinsuke Kikuchi, MD, PhD, Keisuke Kamada, MD, Ai Tochikubo, MD, Daiki Uchida, MD, Atsuhiro Koya, MD, Hiroyuki Kamiya, MD, PhD, and Nobuyoshi Azuma, MD, PhD

**384 Spontaneous Lumbar Artery Injury Resulting in Retroperitoneal Hematoma Mimicking Abdominal Aortic Aneurysm Rupture**

Shingo Nakai, MD, Tetsuro Uchida, MD, PhD, Yoshinori Kuroda, MD, PhD, Atsushi Yamashita, MD, Eiichi Ohba, MD, PhD, Masahiro Mizumoto, MD, Jun Hayashi, MD, Kimihiro Kobayashi, MD, and Tomonori Ochiai, MD

**388 Acute Limb Ischemia in a COVID-19 Patient: A Case Report**

Naohiro Akita, MD, PhD, Takuya Osawa, MD, Hirona Todoroki, MD, and Keisuke Mizuno, MD, PhD

**393 A Case of Late Type Ia Endoleak After Endovascular Aneurysm Sealing Using the Nellix System: Proximal Extension with Triple Chimney and Gutter Endoleak Embolization**

Pietro Volpe, MD, Antonino Alberti, MD, Vittorio Alberti, MD, and Mafalda Massara, MD, PhD

**396 Endovascular Aneurysm Repair for a Patient with Horseshoe Kidney and the Importance of Watershed Sign and Volumetry by Preoperative Contrast-Enhanced Computed Tomography**

Kazuma Handa, MD, Tomohiko Sakamoto, MD, Yumi Kakizawa, MD, Mutsunori Kitahara, MD, PhD, Shinya Fukui, MD, PhD, Yukitoshi Shirakawa, MD, PhD, and Hiroyuki Nishi, MD, PhD

**400 Anterolateral Partial Sternotomy for Treatment of Graft Infection with Fungal Vegetation on the Frozen Elephant Trunk: A Case Report**

Takanori Tsujimoto, MD, Atsushi Omura, MD, PhD, Takeshi Inoue, MD, Syunya Chomei, MD, Mari Hamaguchi, MD, Taishi Inoue, MD, Hidekazu Nakai, MD, Katsuhiro Yamanaka, MD, PhD, and Kenji Okada, MD, PhD

**404 Re-expansion of Thrombosed False Lumen after Aortic Dissection Due to Collateral Retrograde Flow from the Aortic Branches**

Mamoru Hamuro, MD, Senri Miwa, MD, PhD, Kenji Yamamoto, MD, PhD, and Sakae Enomoto, MD, PhD

**407 A Case of Chronic Mesenteric Ischemia: Complete Revascularization Using Multiple Procedures**

Yusuke Yoshimura, MD, Shun-Ichiro Sakamoto, MD, PhD, Atushi Hiromoto, MD, Tomohiro Murata, MD, Kenji Suzuki, MD, PhD, Daisuke Yasui, MD, PhD, Satoshi Mizutani, MD, PhD, and Yosuke Ishii, MD, PhD

**411 Open Abdominal Aortic Repair to Treat Perigraft Seroma after Endovascular Aortic Repair with Endologix AFX2 Endograft**

Masamichi Ozawa, MD, Masaki Hamamoto, MD, PhD, and Taira Kobayashi, MD

**415 Open Repair for Patent Ductus Arteriosus Aneurysm in an Adult**

Yuya Kise, MD, PhD, Yukio Kuniyoshi, MD, PhD, Syotaro Higa, MD, Mizuki Ando, MD, Tatuya Maeda, MD, Hitoshi Inafuku, MD, PhD, and Moriyasu Nakaema, MD

### ANNUAL REPORT

**419 Vascular Surgery in Japan: 2016 Annual Report by the Japanese Society for Vascular Surgery**

The Japanese Society for Vascular Surgery Database Management Committee Member, and NCD Vascular Surgery Data Analysis Team

**439 Reviewers Index**

**441 Authors Index**

**445 Contents of Volume 14, 2021**

**453 Best Cited Articles 2021**

